# Effects of bowel preparation on intestinal bacterial associated urine and faecal metabolites and the associated faecal microbiome

**DOI:** 10.1186/s12876-022-02301-1

**Published:** 2022-05-13

**Authors:** Sam T. R. Powles, Kate I. Gallagher, Leo W. L. Chong, James L. Alexander, Benjamin H. Mullish, Lucy C. Hicks, Julie A. K. McDonald, Julian R. Marchesi, Horace R. T. Williams, Timothy R. Orchard

**Affiliations:** 1 Department of Metabolism, Digestion and Reproduction, Imperial College London, St. Mary’s Hospital, Praed Street, London, W2 1NY UK; 2 Department of Gastroenterology, Imperial College Healthcare NHS Trust, St. Mary’s Hospital, Praed Street, London, W2 1NY UK; 3 MRC Centre for Molecular Bacteriology and Infection, Flowers Building, Imperial College London, London, SW7 2AZ UK

**Keywords:** Bowel cleansing, Colonoscopy, Metabolomics, Metabonomics, Microbiome, Inflammatory bowel disease

## Abstract

**Background:**

Urinary and faecal metabolic profiling have been extensively studied in gastrointestinal diseases as potential diagnostic markers, and to enhance our understanding of the intestinal microbiome in the pathogenesis these conditions. The impact of bowel cleansing on the microbiome has been investigated in several studies, but limited to just one study on the faecal metabolome.

**Aim:**

To compare the effects of bowel cleansing on the composition of the faecal microbiome, and the urine and faecal metabolome.

**Methods:**

Urine and faecal samples were obtained from eleven patients undergoing colonoscopy at baseline, and then at day 3 and week 6 after colonoscopy. 16S rRNA gene sequencing was used to analyse changes in the microbiome, and metabonomic analysis was performed using proton nuclear magnetic resonance (^1^H NMR) spectroscopy.

**Results:**

Microbiomic analysis demonstrated a reduction in alpha diversity (Shannon index) between samples taken at baseline and three days following bowel cleansing (*p* = 0.002), and there was no significant difference between samples at baseline and six weeks post colonoscopy. Targeted and non-targeted analysis of urinary and faecal bacterial associated metabolites showed no significant impact following bowel cleansing.

**Conclusions:**

Bowel cleansing causes a temporary disturbance in bacterial alpha diversity measured in faeces, but no significant changes in the faecal and urine metabolic profiles, suggesting that overall the faecal microbiome and its associated metabolome is resistant to the effects of an induced osmotic diarrhoea.

**Supplementary Information:**

The online version contains supplementary material available at 10.1186/s12876-022-02301-1.

## Synopsis

The intestinal microbiome has been shown to be impacted by bowel cleansing, but the impact on the related metabolome has been much less explored. This study demonstrated a temporary disturbance in faecal microbiome following bowel lavage, but the associated urinary and faecal metabolome remained stable.

## Background

Investigation of the composition and functionality of the gut microbiome is of key interest for a range of gastro-intestinal (GI) diseases [1]. However, there is no standardised best practice regarding choice of sample type (stool or mucosal biopsies), or sample collection methodology and/or processing. It is also recognised that a wide range of external factors may influence the results of analysis. One such major influence is medication, including bowel purgatives such as polyethylene glycol (PEG) solutions, which are given as bowel cleansing prior to colonoscopy [2, 3]; this being of particular relevance when investigating GI disease.

PEG solutions cause a profound osmotic diarrhoea with a high volume lavage rapidly passing through the gastro-intestinal tract, which in turn alters the luminal contents including the microbiota [4, 5]. Effects of bowel cleansing on the intestinal microbiome have been studied [4–10], both to assess whether it can directly cause dysbiosis, and to assess how these vary in health and disease [5].

Results have been inconsistent across different published studies; some have shown a significant reduction in bacterial load [8] and alpha diversity [4, 7, 10] when examining the faecal and colonic mucosal microbiota post bowel cleansing, but this has not been universally demonstrated [5, 6, 9]. In those studies that demonstrate a dysbiosis however, it appears that the composition recovers quickly [5, 8, 10]—likely within 14 days—but the exact timing of this restoration is unclear. Understanding the longevity of these microbial changes may be important in determining study protocols and interpreting results. Of further interest is determining if there are functional metabolic changes (measured in the metabolome) associated with these microbiome perturbations, beyond simply measuring composition. Only one study to date [5] has assessed the metabolic effects of bowel cleansing, using faecal metabonomic analysis.

While faecal metabolic data have been extensively researched in GI conditions [11–16], sample collection can be unappealing for patients, and therefore be more difficult to obtain. Urine is a more readily acquired biosample, and has been shown to demonstrate gut host-microbiota metabolic changes in GI pathology including inflammatory bowel disease (IBD) [17–21] and colorectal cancer [22–25]. No studies have yet been published assessing the impact of bowel cleansing on bacterial associated urinary metabolites or their relationship to the faecal microbiome or metabolome.

This study aimed to compare the effects of bowel cleansing on the faecal microbiome and metabolome at baseline, day 3 and week 6 post colonoscopy.

## Methods

### Experimental design and subjects

This study had ethical approval from Imperial College Healthcare NHS Trust Research and Ethics Committee (Ref: 13/LO/1867). Eleven subjects were recruited from gastroenterology clinics at St. Mary’s Hospital in London who were due to undergo a colonoscopy. Nine participants with no previous history of gastrointestinal disorder were being investigated for a new change in bowel habit and/or diarrhoea; two participants with known ulcerative colitis were being assessed for the activity of inflammatory bowel disease. Subjects were excluded if they had received antibiotics, further purgatives, acid suppressing or immunosuppressive medication within 2 months of sample collection. Written informed consent was obtained from all participants. Detailed dietary and lifestyle data was taken from each subject. A low residue diet containing items such as refined carbohydrates (e.g. white rice), clear soups and white meats, and excluding items high in fibre (e.g. wholemeal bread, fruits with skins) was consumed from two days before the procedure. No solid food was eaten for the final 24 h before the colonoscopy. Participants were given Moviprep^®^ two doses (each dose dissolved in 1 L of water) as a bowel cleanse according to manufacturer guidance.

### Sample collection

32 urine samples and 30 faecal samples were collected from 11 subjects at 3 different time points around their colonoscopy (Additional file 1: Table S1). 9 of these subjects were male, and the mean age was 41 years. The mean BMI was 23.4 kg/m^2^. Two subjects did not provide faecal samples at the last time point, and one of these subjects also did not provide a urine sample at the last time point—however all recruited subjects were included in the final analysis. Baseline (t0) samples were collected 3 days prior to the procedure, and before a low residue diet or bowel purgatives were commenced. Further samples were collected 3 days post procedure (t1), and 6 weeks post procedure (t2). Samples were collected in sterile polypropylene containers and stored in a − 80 °C freezer once received from the subject. MoviPrep^®^ (Macrogol 3350, Sodium sulphate anhydrous, Sodium chloride, Potassium chloride, Ascorbic acid and Sodium ascorbate) was used as bowel preparation in all cases.

### Bacterial DNA extraction and 16S rRNA gene sequencing

DNA extraction was performed using the PowerLyzer PowerSoil DNA Isolation Kit (Mo Bio, Carlsbad, CA, USA). 250 mg of faeces from each sample was used for extraction, and the manufacturer’s instructions were followed. An in-house additional bead beating step [26] was included at speed 8 for 3 min using a Bullet Blender Storm (Chembio Ltd, St Albans, UK). The extracted DNA was then stored at − 80 °C. Illumina’s 16S Metagenomic Sequencing Library Preparation Protocol [27] was used to prepare the sample libraries; the V1-V2 regions of the 16S rRNA gene were amplified using previously-reported primers [28]. These libraries were quantified using the NEBNext Library Quant Kit for Illumina (New England Biolabs, Hitchin, UK). An Illumina MiSeq platform (Illumina Inc., Saffron Walden, UK) was used to perform the sequencing using the MiSeq Reagent Kit v3 (Illumina) and paired-end 300 bp chemistry.

16S rRNA sequencing data was then analysed using the Mothur package (version 2017a, The Mathworks, Inc.; Natwick, MA) following the MiSeq SOP Pipeline [29]. Sequence alignment was performed using the Silva bacterial database, and the Wang method using the ribosomal database project (RDP) database was used for classification of sequences [30]. Operational taxonomic unit (OTU) taxonomies (from phylum to genus) were established using the RDP MultiClassifier Script. The lowest number of reads in a sequenced sample was > 10,000, and subsampling was performed at 10,000 reads per sample. Calculations were performed within Mothur for alpha diversity (Shannon diversity index, H’), and Wilcoxon matched-pairs signed rank test using GraphPad Prism statistical analysis software programme version 8.0.2 was used to assess for statistical significance between time points. Beta diversity was assessed using the non-metric multidimensional scaling (NMDS) plot, and PERMANOVA p-values were generated using the UniFrac weighted distance matrix generated from Mothur, and analysed using the Vegan library within the R statistical package (version 3.3.3) [31]. The Statistical Analysis of Metagenomic Profiles software (STAMP) package [32] was used to assess for statistically significant differences in bacterial composition in the subjects at different time points using White’s non-parametric t-test with Benjamini–Hochberg false discovery rate (FDR).

### Metabolomic analysis

#### Sample preparation

Preparation of urine samples for metabolomic analysis was performed in accordance to standardized protocols. In brief, 540 µL of defrosted urine sample was added to 60 µL of buffer (1.5 M KH_2_PO_4_/D_2_O, 2 mM NaN3 and 0.1% 3-(trimethyl-silyl)propionic acid-d4) (TSP), and centrifuged. 550 µL of the resulting supernatant was transferred into 5 mm diameter NMR tubes. Faecal water was extracted from whole faecal samples by mixing 500 mg of crude stool with two volumes of phosphate buffer saline (PBS) solution, and then vortexing at 2000 Hz for 15 min. These were then centrifuged at 9500 rpm for 20 min at 4 °C, and 600 μl. 400µL of supernatant was transferred to a new Eppendorf, where 100µL of buffer and 250ul D2O were added and the samples centrifuged again. 600µL was transferred to a 5 mm diameter NMR tube. Samples were loaded onto a refrigerated SampleJet robot (Bruker Corporation, Germany) and maintained at 4 °C until analysis. Pooled quality control (QC) samples were also generated for both sample types.

#### Sample analysis

Metabolomic analysis was undertaken using proton nuclear magnetic resonance (^1^H NMR) spectroscopy. All experiments were carried out using an Avance 600 MHz NMR spectrometer (Bruker Biospin), following a standardised protocol [33, 34]. In brief, one dimensional NOESY experiments were carried out on urine and faecal water samples. Spectra were obtained for both sample types at a constant temperature of 300 K. Spectra consisted of 96 K data points, with a spectral width of 20 ppm centred at 4.75 ppm, and pulse width of approximately ~ 13 µs. A double pre-saturation technique was carried out to attenuate the water resonance, harnessing the relaxation delay (4 s), and NOESY mixing time.

### Data analysis for metabolomic samples

Following acquisition, all spectra were phased, calibrated, (using TSP) and baseline corrected automatically in Topspin (version 3.2, Bruker Biospin Ltd.). Spectral data was imported into MATLAB (version 2017a, The Mathworks, Inc.; Natwick, MA) using in-house scripts before spectral regions containing redundant information were removed. These included peaks corresponding to water and TSP, at 4.6–4.85 ppm and − 0.2 to 0.2 ppm respectively. Prior to modelling, all data were aligned using an in-house automatic alignment function. Spectral data was then normalized using a probabilistic quotient approach.

Multivariate statistical analysis was used to investigate differences between study groups. This was performed using SIMCA (version 15, Umetrics, Sweden). Principal components analysis (PCA) was carried out using univariate scaling to allow for the identification of any outliers and clustering based on principal components.

For targeted analysis, peak integral values for selected metabolites were obtained using an in-house Matlab script [26]. Using the integral values, a univariate statistical approach was used to compare the relative amount of metabolites of interest between the three time points. GraphPad Prism statistical analysis software programme version 8.0.2 was used to perform a Wilcoxon matched-pairs signed rank test between time points t0 and t1, t0 and t2, and t1 and t2. A Bonferroni calculation was used to correct for multiple comparisons. Urine metabolites [17, 19–25, 35, 36] and faecal metabolites [11–13, 37, 38] were selected for targeted analysis if they are produced by intestinal bacterial metabolism or host-bacterial co-metabolism, and in previous studies have been shown to be important in GI disease.

## Results

### Faecal 16 s RNA sequencing

**Fig. 1:**
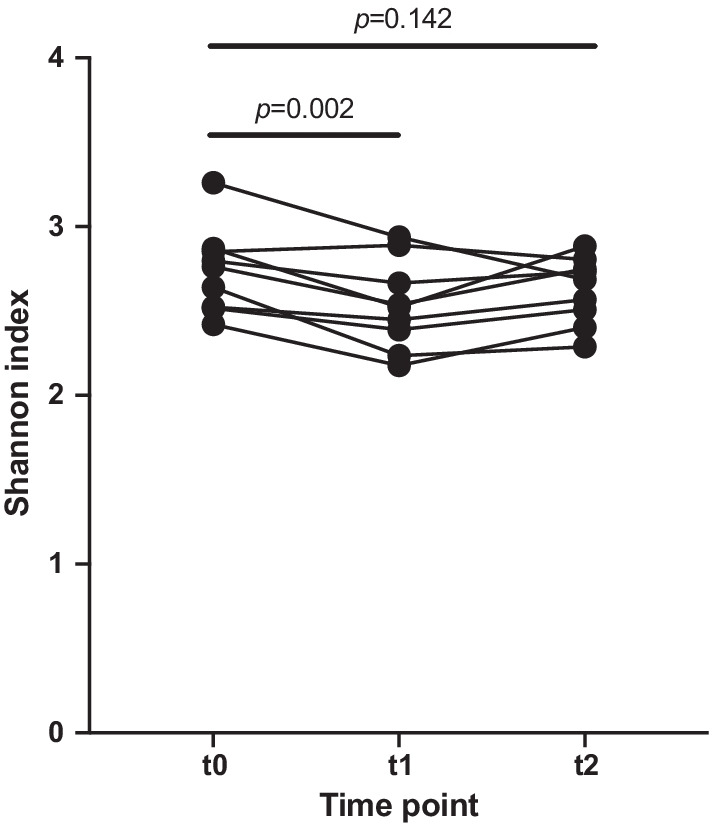
Faecal microbiota alpha diversity as measured by the Shannon index at baseline (t0), 3 days after bowel preparation (t1), and 6 weeks after bowel preparation (t2)

31 faecal samples were collected, which included 11 samples at baseline (t0), 11 at three days post colonoscopy (t1), and 9 at six weeks post procedure (t2). Analysis of the alpha diversity between baseline (t0) and 3 days post procedure (t1) showed that bowel preparation caused a significant decrease (*p* = 0.002) in the Shannon index of the bacteria present (Fig. 1). There was no significant change between baseline and 6 weeks post bowel cleansing (t0 and t2).

**Fig. 2:**
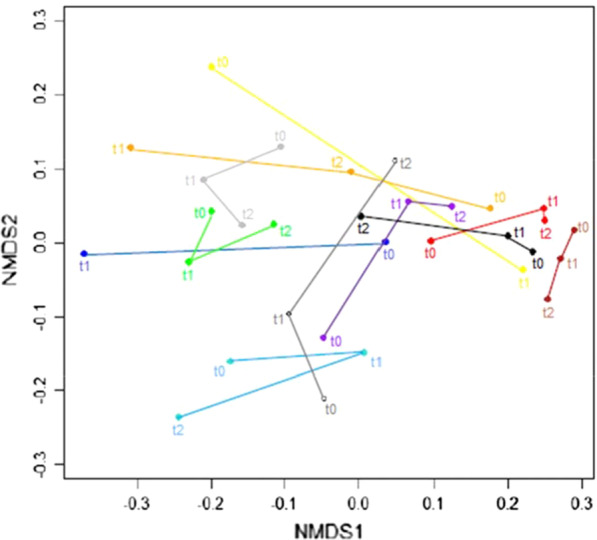
NMDS plot of microbial composition of subjects at differing time points. t0 = baseline, t1 = 3 days post colonoscopy, and t2 = 6 weeks post colonoscopy. Lines and labels have been added to figure to clarify subjects and time points

Compositional changes between samples (beta diversity) were analysed using non-metric multidimensional scaling (NMDS) plots measuring weighted Unifrac distances—see Fig. 2. There were no statistically significant differences when comparing samples across different time points permutational multivariate analysis of variance (PERMANOVA).

**Fig. 3:**
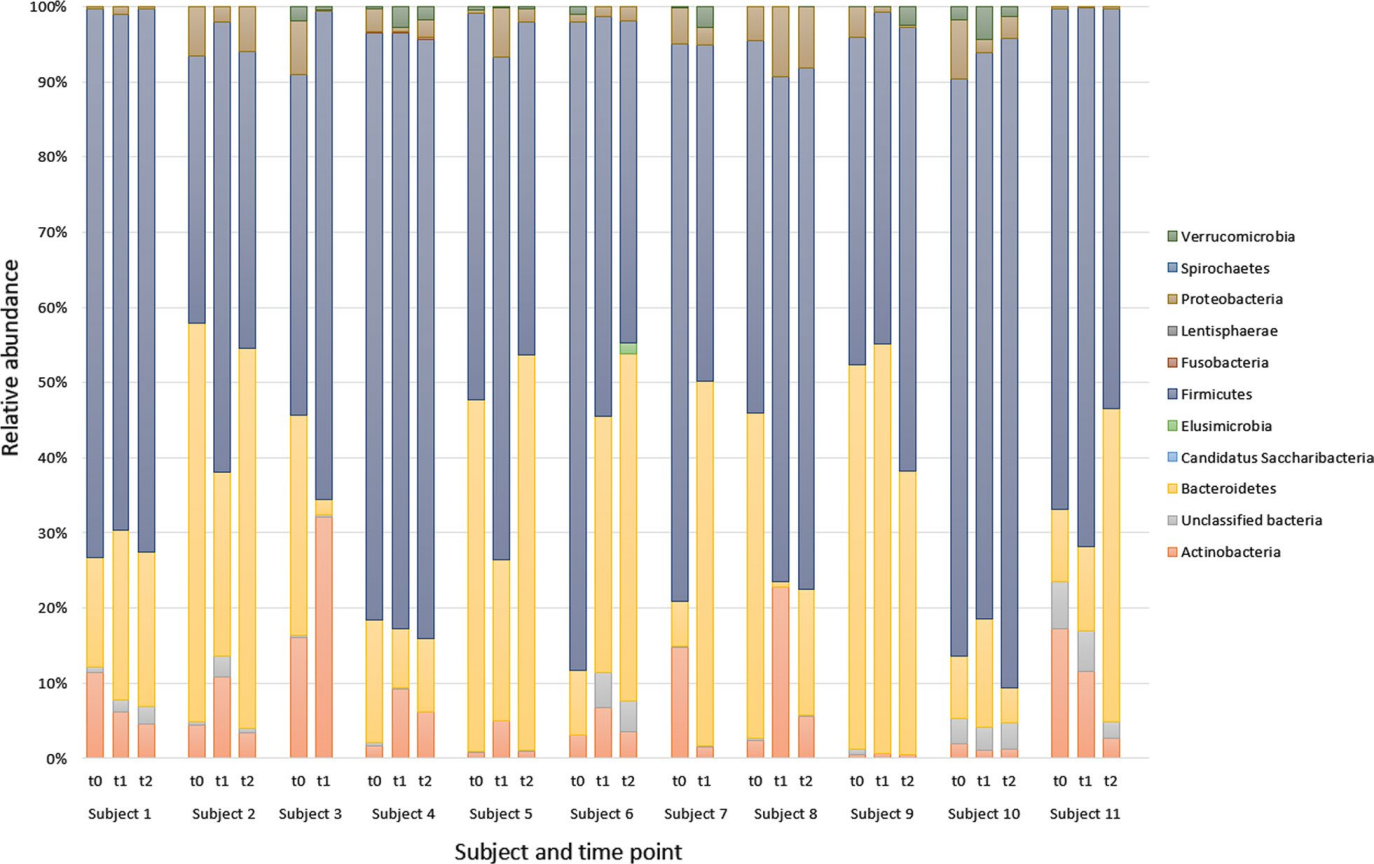
Percentage bar graph showing the relative proportion of bacterial phyla in each sample. Samples are grouped within subjects and in order of collection time; at baseline (t0), 3 days after bowel preparation (t1), and 6 weeks after bowel preparation (t2)

Taxanomic analysis at phylum, class, order, family, and genus levels was performed using the STAMP software package, and there was no significant difference in composition of bacteria between samples taken at baseline, 3 days post colonoscopy, and 6 weeks post procedure—see Fig. 3.

### Urine metabonomic analysis

Metabonomic analysis was performed on 32 urine samples which were collected at the same time as the faecal samples. This included 11 baseline samples (t0), 11 collected at 3 days post procedure (t1), and 10 collected at 6 weeks post procedure (t2). Detailed lifestyle and dietary data obtained from the study subjects showed no significant differences between time points—see Additional file 1: Table S2.

**Fig. 4:**
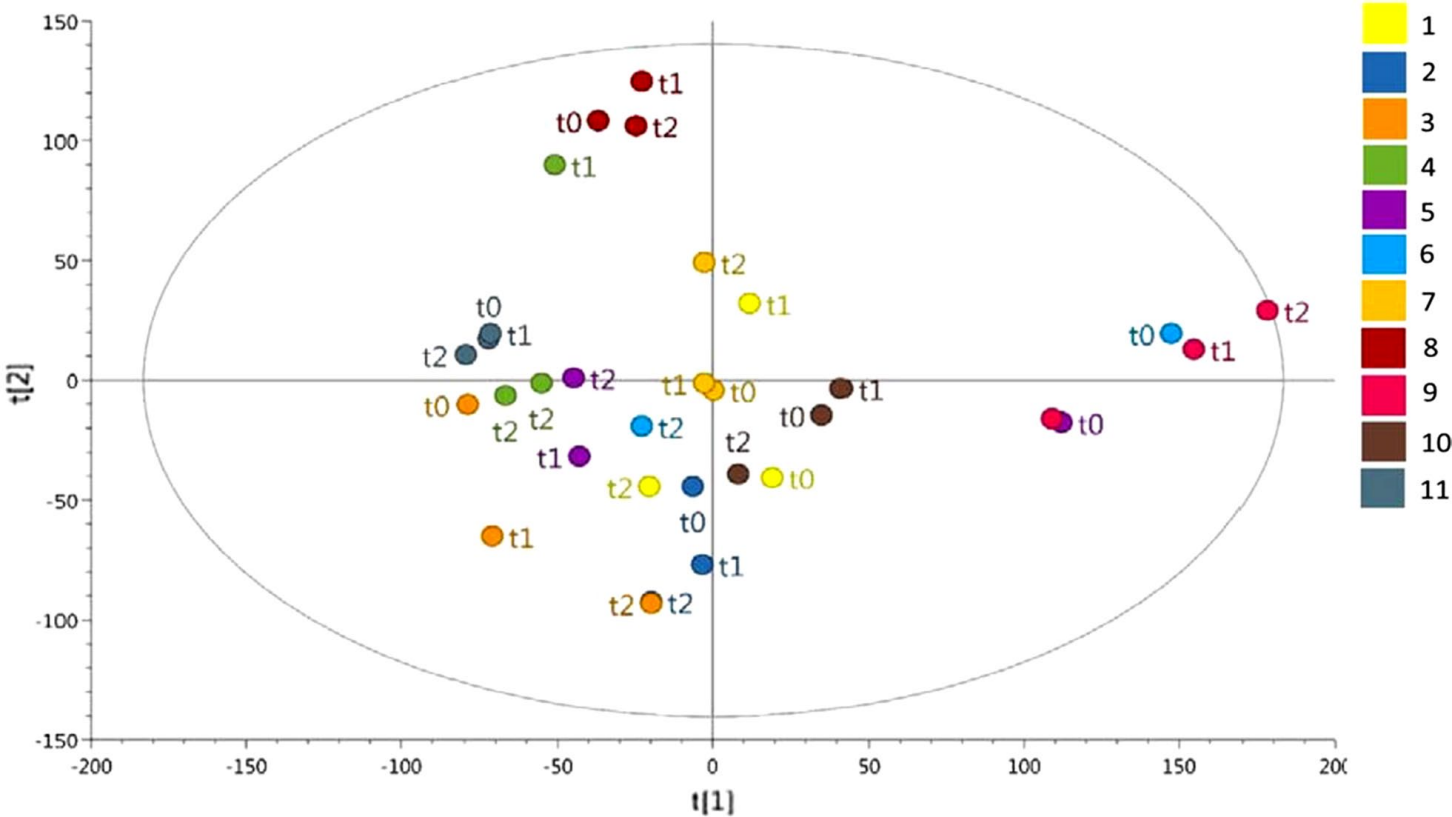
Principal component analysis (PCA) scores plot of urine metabolic profiles of 11 subjects, with samples at baseline, 3 days post procedure, and 6 weeks post procedure. Samples from the same subject are plotted in the same colour, with participant number as shown in the key; sampling time points are also shown for each participant, i.e. baseline (t0), 3 days after bowel preparation (t1), and 6 weeks after bowel preparation (t2). This figure shows intra-subject clustering in samples from 9 out of 10 subjects over the 3 time points

Unsupervised multivariate analysis using principal component analysis (PCA) of the urine samples at each time point was performed—see Fig. 4. In 9 out of the 11 subjects, there was intra-subject clustering of samples despite the use of bowel cleansing.

**Fig. 5:**
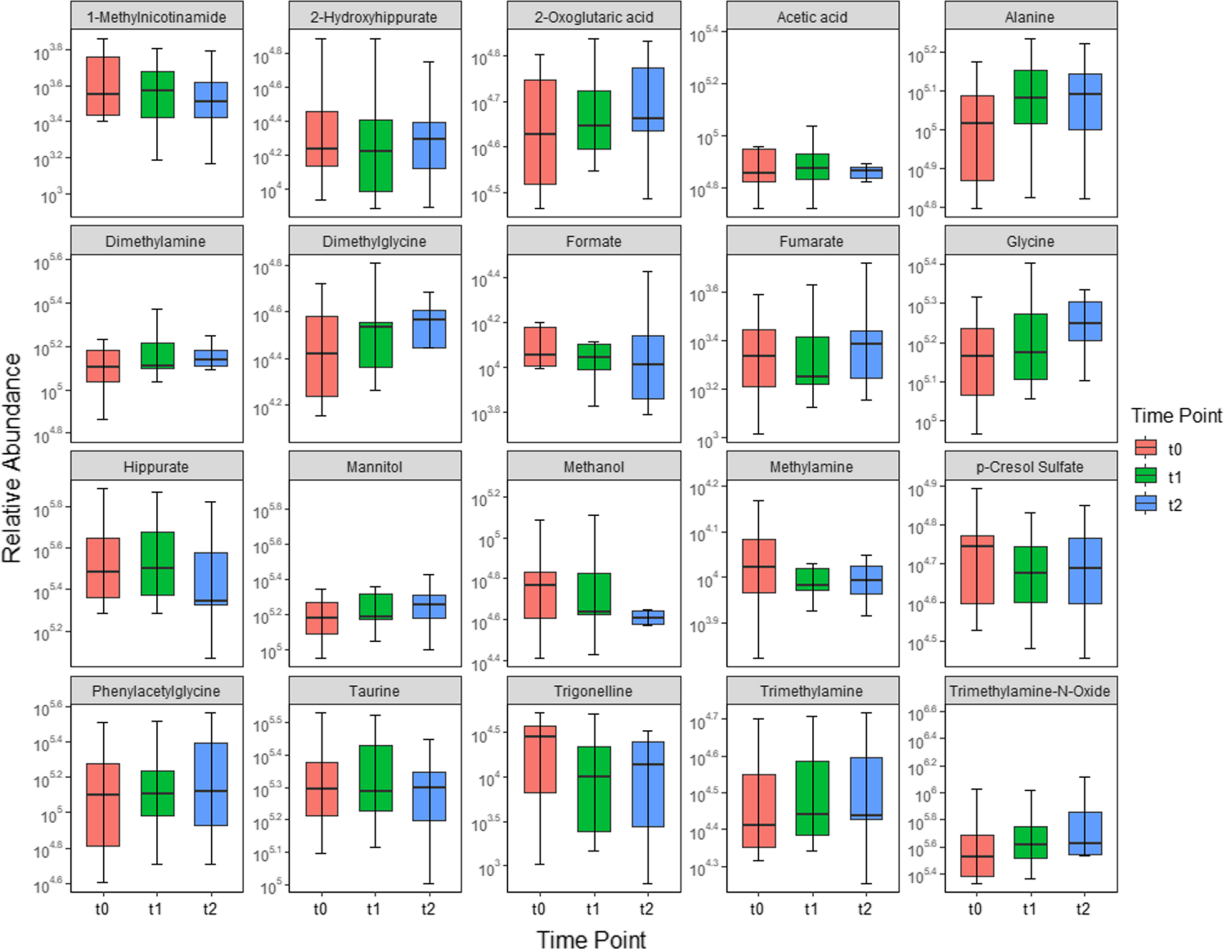
Bar plot graphs showing median relative quantity of each urine metabolite at three time points; at baseline (t0), 3 days after bowel preparation (t1), and 6 weeks after bowel preparation (t2)

Targeted analysis of 20 urine metabolites revealed that alanine excretion was higher at 3 days and 6 weeks post bowel cleansing in comparison to baseline (*p* value 0.005, and 0.020 respectively) but these differences were not significant after correcting for multiple comparisons—see Fig. 5 and Additional file 1: Table S3.

### Faecal metabonomic analysis

Metabonomic analysis was performed on ten faecal samples at baseline (t0), and ten samples 3 days post colonoscopy (t1). Unsupervised multivariate analysis of faecal water metabolites was performed using Principal Component Analysis—see Additional file 1: Figure S1. This showed that in 9 out of 10 subjects, there was intra-subject clustering of samples despite the use of bowel cleansing. Targeted analysis was performed on 10 metabolites. Butyrate was higher at 3 days in comparison to baseline (*p* value = 0.027) but this difference was not significant after correcting for multiple comparisons—see Additional file 1: Table S4.

## Discussion

This is a novel study combining urinary and faecal metabolic data with microbiomic changes to assess the effect of bowel cleansing used pre-colonoscopy.

Stool analysis showed a reduction in microbiome ecological indices, with a significant decrease in the Shannon index (*p* = 0.002) following bowel preparation. In a similar study, Gorkiewicz et al. [4] demonstrated a reduction in richness following three days of PEG administration, and this change trended towards recovery but remained significantly lower than baseline one week after bowel cleansing. A trend towards reduced Shannon index was also shown by Shobar et al. [10] within one week post bowel cleansing. However, four other studies did not show a change in Shannon index following bowel cleansing [5, 6, 8, 9].

Inter-subject variability was more marked than intra-subject variability in all but three patients following bowel preparation, and there were no statistically significant changes in beta diversity between samples across the different time points. Similar beta diversity analysis published by Nagata et al. [5], using principal coordinated analysis (PCoA) plot of weighted UniFrac distances, also showed individuals who had received bowel cleansing clustered together, rather than clustering at time points. A study by Shobar et al. [10] also did not show a statistically significant difference in weighted Unifrac distances in faecal samples pre- and post-bowel cleansing, but did show a difference in unweighted distances, potentially suggesting that rarer species were affected more by bowel cleansing.

Taxonomic composition analysis showed no statistically significant changes from phylum to genus level following bowel cleansing. Negata et al. [5] showed no changes in the relative abundance at phyla level in eight patients receiving bowel cleansing, and in genera with a > 1% relative abundance. A larger study by O'Brien et al. [9] showed no consistent findings in taxonomic composition in 15 patients undergoing bowel lavage. Another study by Shobar et al. [10] showed a reduction in *Bacteroidetes* in ten healthy controls and eight IBD patients receiving bowel preparation, along with changes at other taxonomic levels including a reduction in the *Clostridiales* order. A reduction in *Firmicutes* and an increase in *Proteobacteria* was reported in a study by Drago et al. [6] at phyla level, who also showed a reduction in the *Clostridia* class. Discrepancies between previous studies [4–6, 8–10, 39] assessing the impact of bowel cleansing on the faecal microbiota are likely in part due to the differing study designs, time point analysis, analytical techniques and generally small sample sizes.

The effects of bowel cleansing on intestinal bacterial-associated urine metabolites was investigated in this study, measuring selected targeted metabolites which have been previously demonstrated to alter in GI diseases including IBD and colorectal cancer [17, 19–23, 25, 35, 36]. These included formate, hippurate, p-cresol sulphate, and alanine [17, 21]. Formate, produced both endogenously and from intestinal fermentation, has been associated with the *Enterobacteriaceae* phylum, and particularly *Escherichia coli* [40, 41]. Hippurate, a product of host and commensal co-metabolism of dietary aromatic compounds, has been shown in several studies to have reduced excretion in IBD [17, 19–21], and to have a positive association with *Clostridia* species [42]. Bacterial fermentation of tyrosine in the colon produces p-cresol sulphate, and its production has also been associated with *Clostridia* species [43]. Beta-alanine, an isomer of alanine, is a non-protein amino acid obtained from dietary muscle protein and additionally sourced from intestinal *Escherichia coli* [44]. Trimethylamine N-oxide (TMAO) has been associated with the development of colorectal cancer [16, 45]. TMAO is reduced to trimethylamine (TMA) by predominantly *Enterobacteriaceae* in the gut [46].

There were no significant changes in any of the urinary metabolites that were measured following bowel cleansing (with the exception of alanine, although this was not significant when corrected for multiple comparisons). Unsupervised multivariate analysis showed that in nine out of eleven patients, subject clustering was greater than time point clustering irrespective of bowel cleansing, suggesting stability of bacterial associated urinary metabolites despite alterations in the intestinal faecal microbiome. Untargeted multivariate analysis of faecal metabolites showed that at day 3 post bowel cleansing subject clustering was still present in 9 out of 10 subjects, and targeted univariate analysis showed higher butyrate excretion at day 3, although statistical significance for this change in butyrate was lost once corrected for multiple comparisons. A recent study by Nagata et al. [5] showed an immediate faecal metabolic perturbation in the first catch samples following bowel purgative ingestion, but subject clustering and changes in specific metabolites were restored at day 14 (the next sampling point in this study).

A stable metabolome despite a transient change in one aspect of the intestinal microbiome composition (alpha diversity) may suggest functional redundancy, where despite loss of specific bacterial species, the overall metabolic processes are preserved by cross-compensation by other bacteria [47]. These results suggest that in patients undergoing bowel preparation for colonoscopy, PEG may potentially confound results of microbiome studies, but appears less likely to impact studies of microbiomic function.

Several limitations were present in this study, including a relatively small number of subjects—although this was a similar cohort size to previous studies assessing the effects of bowel cleansing. Although all the colonoscopy examinations showed no significant active pathology, all subjects were recruited from gastroenterology clinic and reporting gastrointestinal symptoms, and so there was a mixture of subjects with quiescent ulcerative colitis and irritable bowel syndrome within the study subjects. More than 80% of the study participants were male, meaning that our results might not be reflective of a cohort with a lower proportion of male subjects. The week 6 sample cohort was incomplete, with two subjects not giving faecal samples, and one subject not giving a urine sample. Moreover, faecal metabolomics was not performed at week 6 owing to a lack of faecal material available for the analysis. The initial post-intervention sample time point was taken three days after bowel cleansing, and so more immediate perturbations in metabolites and the faecal microbiome may have been missed. NMR spectroscopy was used as the analytical platform, which gives a good overall qualitative and relative quantitative assessment of metabolites, but is less sensitive than mass spectrometry [48].

In conclusion, this study shows that bowel cleansing causes a temporary disturbance in bacterial alpha diversity measured in faeces, but no significant changes in the urine and faecal metabolome. This suggests overall the faecal microbiome and its associated metabolome is resistant to the effects of an induced osmotic diarrhoea.

## Supplementary Information

**Additional file 1.**Supplementary Tables 1 to 4, and Supplementary Figure 1.

## Data Availability

The datasets generated during and/or analysed during the current study are not publicly available at this time but are available from the corresponding author on reasonable request.

## Abbreviations

^1^H NMR: Proton nuclear magnetic resonance
BMI: Body mass index
CD: Crohn’s disease
GI: Gastrointestinal
NMDS: Non-metric multidimensional scaling
PEG: Polyethylene glycol
t0: Time point 0—baseline
t1: Time point 1—3 days post colonoscopy
t2: Time point 2—6 weeks post colonoscopy
UC: Ulcerative colitis
