# Supplementary material for: Effects of bowel preparation on intestinal bacterial associated urine and faecal metabolites and the associated faecal microbiome
Source: BMC Gastroenterol. 2022 May 13;22:240. doi: 10.1186/s12876-022-02301-1 (PMC9101932; doi:10.1186/s12876-022-02301-1)
Supplement: Supplementary file 1 — Additional file 1.Supplementary Tables 1 to 4, and Supplementary Figure 1. [file 12876_2022_2301_MOESM1_ESM.docx]

Supplementary Data:

Effects of bowel preparation on intestinal bacterial associated urine and faecal metabolites and the associated faecal microbiome.

**Supplementary Table 1. Metadata of recruited patients.**

| **Sbj.** | **Age** | **Sex** | **Ethnicity** | **BMI** | **Co-morbidities** | **Medications** | **Indication** | **Colonoscopy report** | **Histology** | **Diagnosis** | **Samples obtained**** |
| --- | --- | --- | --- | --- | --- | --- | --- | --- | --- | --- | --- |
| 1 | 41 | M | Caucasian | 27.2 | Ulcerative colitis | Pentasa | Assess IBD | Patchy erythema distally. | Within normal limits | UC | Faeces for 16S: t0, t1, t2  Faeces for NMR: t0, t1  Urine for NMR: t0, t1, t2 |
| 2 | 63 | M | Caucasian | 25.6 | Ulcerative colitis,  type 2 diabetes mellitus | Metformin | Assess IBD | UC mild activity in distal 20cm | Quiescent colitis in rectum | UC | Faeces for 16S: t0, t1, t2  Faeces for NMR: t0, t1  Urine for NMR: t0, t1, t2 |
| 3 | 31 | M | Caucasian | 19.7 | Nil | Nil | CIBH* | Normal | Nil taken | IBS | Faeces for 16S: t0, t1  Faeces for NMR: t0, t1  Urine for NMR: t0, t1, t2 |
| 4 | 28 | M | Caucasian | 34.6 | Nil | Nil | Diarrhoea | Normal | Within normal limits | IBS | Faeces for 16S: t0, t1, t2  Faeces for NMR: t0, t1  Urine for NMR: t0, t1 |
| 5 | 24 | M | Caucasian | 18.5 | Nil | Nil | Diarrhoea | Normal | Within normal limits | IBS | Faeces for 16S: t0, t1, t2  Faeces for NMR: t0, t1  Urine for NMR: t0, t1, t2 |
| 6 | 45 | F | Caucasian | 21.3 | Nil | Nil | CIBH | Normal | Nil taken | IBS | Faeces for 16S: t0, t1, t2  Faeces for NMR: t0, t1  Urine for NMR: t0, t1, t2 |
| 7 | 47 | M | Caucasian | 23.6 | Hypertension | Candesartan | CIBH | Normal | Nil taken | IBS | Faeces for 16S: t0, t1  Faeces for NMR: t0, t1  Urine for NMR: t0, t1, t2 |
| 8 | 31 | M | Black | 23.3 | Nil | Nil | CIBH | Normal | Nil taken | IBS | Faeces for 16S: t0, t1, t2  Faeces for NMR: t0, t1  Urine for NMR: t0, t1, t2 |
| 9 | 40 | F | Caucasian | 20.7 | Nil | Nil | Diarrhoea | Normal | Within normal limits | IBS | Faeces for 16S: t0, t1, t2  Faeces for NMR: t0, t1  Urine for NMR: t0, t1, t2 |
| 10 | 38 | M | Caucasian | 20.1 | Nil | Nil | CIBH | Normal | Nil taken | IBS | Faeces for 16S: t0, t1, t2  Faeces for NMR: t0, t1  Urine for NMR: t0, t1, t2 |
| 11 | 58 | M | Caucasian | 22.6 | Nil | Nil | CIBH | Normal | Nil taken | IBS | Faeces for 16S: t0, t1, t2  Faeces for NMR: nil  Urine for NMR: t0, t1, t2 |

*CIBH – change in bowel habit, **this indicates for each of the analyses whether samples were obtained for baseline (t0), day 3 post colonoscopy (t1), and 6 weeks post colonoscopy (t2).

**Supplementary Table 2 – Lifestyle and dietary comparisons between different time points**

|  | Time point 0  (yes : no) | Time point 1  (yes : no) | Time point 2  (yes : no) | p value^†^ |
| --- | --- | --- | --- | --- |
| Vegetarian | 0 : 11 | 0 : 11 | 0 : 11 | 1 |
| Meat^*^ | 7 : 4 | 8 : 3 | 7 : 4 | 0.872 |
| Fish^*^ | 5 : 6 | 4 : 7 | 4 : 7 | 0.881 |
| Cherries^*^ | 2 : 9 | 2 : 9 | 3 : 8 | 0.834 |
| Cheese^*^ | 6 : 5 | 5 : 6 | 7 : 4 | 0.693 |
| Grapefruit^*^ | 0 : 11 | 2 : 9 | 1 : 10 | 0.333 |
| Liquorish^*^ | 0 : 11 | 0 : 11 | 0 : 11 | 1 |
| Walnuts^*^ | 2 : 9 | 0 : 11 | 1 : 10 | 0.333 |
| Vanilla^*^ | 2 : 9 | 0 : 11 | 2 : 9 | 0.320 |
| Yoghurt^*^ | 4 : 7 | 3 : 8 | 5 : 6 | 0.675 |
| Berries^*^ | 3 : 8 | 3 : 8 | 2 : 9 | 0.848 |
| Carbonated drinks^*^ | 2 : 9 | 4 : 7 | 4 : 7 | 0.563 |
| Coffee^*^ | 6 : 5 | 7 : 4 | 6 : 5 | 0.883 |
| Milk^*^ | 8 : 3 | 7 : 4 | 7 : 4 | 0.873 |
| Tea^*^ | 3 : 8 | 5 : 6 | 4 : 7 | 0.675 |
| Herbal tea^*^ | 0 : 11 | 0 : 11 | 1 : 10 | 0.357 |
| Herbal remedies^*^ | 0 : 11 | 0 : 11 | 0 : 11 | 1 |
| Alcohol^*^ | 5 : 6 | 4 : 7 | 5 : 6 | 0.883 |
| Smoker | 1 : 10 | 1 : 10 | 1 : 10 | 1 |
| Exercise^*^ | 5 : 6 | 4 : 7 | 5 : 6 | 0.883 |

**Supplementary Table 2.**  Dietary and lifestyle comparison of study subjects at time point 0 (baseline), time point 1 (3 days post procedure), and time point 2 (6 weeks post procedure) . ^*^Consumed within 24 hours of sample collection. ^†^analysed by Chi-squared test. Significant p values (p < 0.05).

**Supplementary Table 3. Univariate analysis comparing urine metabolites between time points.**

| **Metabolite** | **t0 vs t1** | **t0 vs t2** | **t1 vs t2** |
| --- | --- | --- | --- |
| 1-methylnicotinamide | 0.577 | 0.057 | 0.492 |
| 2-hydroxyhippurate | 0.240 | 0.846 | 0.923 |
| 2-oxoglutaric acid | 0.32 | 0.492 | 0.432 |
| acetic acid | 0.465 | 0.492 | 0.929 |
| alanine | 0.005 | 0.020 | 0.770 |
| p-cresol sulphate | 0.365 | 0.846 | 0.922 |
| dimethylamine | 0.831 | 0.695 | 0.625 |
| dimethylglycine | 0.240 | 0.625 | 0.922 |
| formate | 0.765 | 0.322 | 0.695 |
| fumurate | 0.700 | 0.275 | 0.922 |
| glycine | 0.175 | 0.131 | 0.492 |
| hippurate | 0.966 | 0.770 | 0.557 |
| mannitol | 0.413 | 0.557 | 0.696 |
| methanol | 0.765 | 0.322 | 0.695 |
| methylamine | 0.520 | 0.770 | 0.375 |
| phenyacetylglutamine (PAG) | 0.638 | 0.322 | 0.492 |
| timethylamine | 0.831 | 0.432 | 0.770 |
| taurine | 0.700 | 0.625 | 0.769 |
| trigonelline | 0.413 | 0.193 | 0.432 |
| Trimethylamine  N-oxide (TMAO) | 0.898 | 0.625 | 0.846 |

**Supplementary Table 3.** Changes in urine metabolites between baseline (t0), day 3 post bowel preparation (t1), and 6 weeks post procedure (t2). Wilcoxon matched-pairs signed rank test to assess for statistical significance. p value figures are shown before correction for multiple comparisons with a Bonferroni calculation. Prior to correction for multiple comparisons, alanine excretion was higher in day 3 and week 6 samples compared to baseline.

**Supplementary Figure 1. Unsupervised multivariate analysis of faecal water metabolites at each time point.**


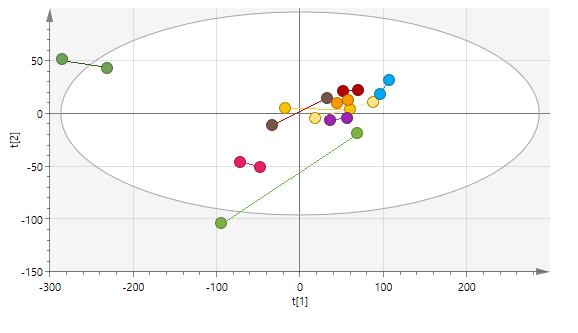


**Supplementary Figure 1.** PCA scores plot of faecal metabolic profiles of 11 subjects, with samples at baseline and 3 days post procedure. Samples from the same subject are plotted in the same colour. Lines have been added to this figure to link time points between samples from the same subject. This figure shows intra-subject clustering in samples from 9 out of 10 subjects over the time points.

**Supplementary Table 4. Univariate analysis comparing faecal water metabolites between time points.**

| **Metabolite** | **p value - t0 vs t1** |
| --- | --- |
| acetate | 0.084 |
| butyrate | 0.027 |
| propionate | 0.232 |
| lactate | 0.193 |
| methylamine | 0.695 |
| glutamine | 0.065 |
| alanine | 0.557 |
| taurine | 0.193 |
| valine | 0.846 |
| alpha hydroxybutyrate | 0.695 |

**Supplementary Table 4.** Changes in faecal water metabolites between baseline (t0), day 3 post bowel preparation (t1), and 6 weeks post procedure (t2). Wilcoxon matched-pairs signed rank test to assess for statistical significance. p value figures are shown before correction for multiple comparisons with a Bonferroni calculation. Prior to correction for multiple comparisons, butyrate excretion was higher in day 3 samples compared to baseline (p = 0.027).
